# ID 145 - Eficácia e Segurança da Toxina Botulínica para Tratamento de Bexiga Hiperativa de Etiologia Neurogênica em Adultos e Idosos

**DOI:** 10.5327/2237-9622.2025.v34s1.145

**Published:** 2025-11-25

**Authors:** Maria Paula Custódio Silva, Maria Paula Custódio Silva, Karoline Faria de Oliveira, Giovani Luiz De Santi, Sérgio Antônio Zullo, Daniele Oliveira dos Santos, Priscila Andreja Oliveira, Nylze Helena Guillarducci Rocha, Andréa Silva Dutra Tirones, Luana Pereira Cunha Barbosa, Rhaíssa Fernandes Batista, Saulo Pereira da Costa, Valter Paulo Neves Miranda

**Keywords:** incontinência urinária, toxinas botulínicas tipo A, avaliação de tecnologias

## Abstract

**Introdução:**

A bexiga neurogênica é uma condição de saúde complexa e debilitante, caracterizada pelo mal funcionamento do sistema urinário inferior devido a alterações neurológicas. O tratamento medicamentoso para a bexiga neurogênica visa aliviar os sintomas e melhorar a qualidade de vida dos pacientes. Nesse contexto, a toxina botulínica (TXB) tem sido empregada para aqueles pacientes que não respondem aos antimuscarínicos, por se tratar de um agente bloqueador neuromuscular que causa paralisia neuromuscular flácida transitória.

**Objetivos:**

Avaliar a eficácia e a segurança do uso da TXB tipo A no tratamento da bexiga hiperativa de etiologia neurogênica, refratária a tratamento com anticolinérgicos, em adultos e idosos.

**Método:**

Revisão sistemática com metanálise, registro PROSPERO CRD42024569413. Foram analisados Ensaios Clínicos Randomizados que compararam a intervenção (TXB Tipo A) com placebo, adultos e/ou idosos, com bexiga hiperativa de etiologia neurogênica que tiveram condição refratária ao tratamento com anticolinérgicos. Não houve restrição quanto ao tamanho da amostra, idioma, tempo de seguimento ou data de publicação. Foi realizada uma busca sistematizada nas bases de dados PubMed, Embase, LILACS (BVS) e Cochrane Library. A seleção e a extração dos dados foi realizada por duplas independentes de pesquisadores com posterior consenso realizado por um outro pesquisador independente. Foi realizada avaliação do risco de viés dos estudos incluídos utilizando o RoB2 e a certeza da evidência verificada pelo GRADE2. A análise para metanálise foi conduzida pelo software Review Manager 5.4.

**Resultados:**

De um total de 821 documentos encontrados, foram incluídos oito artigos. A maioria dos estudos (87,5%) avaliou como desfechos principais o número de episódios de incontinência (IU), a capacidade máxima cistométrica (CMC) e a pressão máxima de contração do músculo detrusor (PMD). A avaliação do risco de viés evidenciou que apenas um estudo apesentou alto risco, cinco algumas preocupações e dois baixo risco para os desfechos melhora clínica, qualidade de vida (QV) e eventos adversos. A segurança foi comprovada com base no baixo número de efeitos adversos graves. Quanto à comparação da TXB com o placebo nos desfechos primários, para a IU foi constatada, pela metanálise, diminuição da diferença média (DM) diária de -6,78 de episódios miccionais (IC 95% -8,21 a -5,35, I²: 98%, p<0,001). Para a CMC, a metanálise mostrou aumento da DM de 154,93 ml (IC 95% 147,10 a 162,75, I²: 89%, p<0,001). Assim como houve redução na PDM, DM de -28,07 cmH2O (IC 95% -29,43 a -26,70, I²: 82%, p<0,001). Por fim, observou-se melhora na QV, com aumento da DM dos escores do I-QOL de 15,32 pontos (IC 95% 14,45 a 16,19, I²: 73%, p=0,005). Na análise de subgrupo, constatou-se que a CMC e a PMD apresentaram valores mais ajustados e menos heterogêneos (I²<70%, p>0,01) para dosagem de TXB entre 100 e 200 U. A qualidade da evidência, pelo GRADE, foi considerada baixa para a IU, moderada para os desfechos hemodinâmicos (CMC e PMD) e alta para QV.

**Conclusão:**

As evidências científicas dos desfechos foram melhores para medidas urodinâmicas e qualidade de vida. As dosagens entre 100 U e 200 U mostraram-se mais eficientes e mais consistentes, assim como também foi observado em outro estudo. Conclui-se que a TXB foi segura e eficaz no tratamento de pacientes adultos e idosos com bexigas hiperativas neurogênicas, refratárias a anticolinérgicos. No entanto, sugere-se monitoramento da manutenção de sua eficácia e segurança.

